# Elevated COX-2 Expression Promotes Angiogenesis Through EGFR/p38-MAPK/Sp1-Dependent Signalling in Pancreatic Cancer

**DOI:** 10.1038/s41598-017-00288-4

**Published:** 2017-03-28

**Authors:** Hai Hu, Ting Han, Meng Zhuo, Lei-lei Wu, Cuncun Yuan, Lixia Wu, Wang Lei, Feng Jiao, Li-Wei Wang

**Affiliations:** 1 Department of Medical Oncology, Shanghai General Hospital, Shanghai Jiao Tong University School of Medicine, 650 New Songjiang Road, Shanghai, 201620 China; 2 Shanghai Key Laboratory of Pancreatic Disease, Shanghai, 201620 China; 3 School of Life Sciences and Biotechnology, Shanghai Jiao Tong University, Shanghai, 201620 China; 4 Department of Pathology, Shanghai General Hospital, School of Medicine, Shanghai Jiao Tong University, Shanghai, 201620 P.R. China

## Abstract

Cyclooxygenase-2 (COX-2) was stated to be overexpression in various human malignancies associating with angiogenesis, metastasis and chemoresistence. Pancreatic ductal adenocarcinoma (PDAC) is a lethal disease displaying many of these characteristics. A common abnormality of PDAC is overexpression of specificity protein-1 (Sp1), which was said to correlate with malignant phenotypes of human cancers. Using RNA-seq data from The Cancer Genome Atlas (TCGA), we found that Sp1 expression was positively correlated with that of COX-2 in PDAC, and that the inhibition or overexpression of Sp1 in PDAC cells leads to decreased or elevated COX-2 expression. Luciferase reporter gene and chromatin immunoprecipitation (ChIP) assays revealed that elevated transcription of COX-2 requires Sp1 binding to sequence positions around −245/−240 of COX-2 promoter. Activated epidermal growth factor receptor (EGFR) and downstream p38 mitogen-activated protein kinase (p38-MAPK) were also profoundly altered in PDAC. The inhibition of EGFR/p38-MAPK signaling resulted in reduced Sp1 activation, decreased COX-2 and vascular endothelial growth factor (VEGF) expression. Thus, Sp1 could transcriptionally activate COX-2 expression in a process relies on activated EGFR/p38-MAPK signaling. Finally, we found that the inhibition of COX-2 leads to decreased angiogenesis in a process dependent on VEGF, which link COX-2 to angiogenesis in PDAC.

## Introduction

Pancreatic ductal adenocarcinoma (PDAC) is a devastating disease with the worst prognosis among all major human malignancies. The long-term prognosis of the disease remains poor with a 5-year survival rate of <5% and a median survival time of 6 months^1^, a frustrating situation that has not been improved for decades. The major causes for the lack of progress in improving prognosis include this cancer’s high propensity for early distant metastasis and chemoresistance^2^. Moreover, the molecular and cellular mechanisms underlying these events remain elusive.

In recent years, researchers have gradually acknowledged the pro-tumoural properties of chronic inflammation^3^. COX-2, a key enzyme implicated in inflammation, had been reported to be elevated in major human malignancies. In addition, COX-2 has been implicated in angiogenesis, chemoresistence and distant metastasis in multiple cancers^4^. In clinical settings, elevated COX-2 expression correlated with a decreased overall survival (OS), and targeting COX-2 could have substantial survival benefits for patients^5, 6^. Specifically, elevated COX-2 has also been reported in cases of PDAC, and the inhibition of COX-2 by celecoxib resulted in reduced tumour growth^7^. Despite these advancements, no reports to date had presented a clear illustration as to how COX-2 is regulated during PDAC progression.

Specificity protein (Sp1) is a nuclear transcription factor located in the nucleus. As stated, Sp1 expressed ubiquitously in all cells of an individual and is responsible for cell proliferation, differentiation and apoptosis^8, 9^. Structurally, there are 3 zinc fingers in the C-terminal region of Sp1, which bind to the GC box of target genes and activate their expression^10^. Since COX-2 promoter is also rich in GC boxes^11^, we hypothesized that these two pro-tumoural factors may be related, and that their mutually high expression has synergistic effects on promoting PDAC progression.

In the present study, we investigated the biological significance and the regulation of COX-2 in PDAC. We found that the elevated expression levels of Sp1 and COX-2 are positively correlated in PDAC cells. Our data demonstrated that Sp1 could bind directly to COX-2 promoter and activate it expression in a manner dependent on EGFR/p38-MAPK signalling. In addition, we also revealed that elevated COX-2 could promote angiogenesis in a VEGF-dependent manner.

## Results

### Correlated expression of Sp1 and COX-2 in clinical PDAC tissues

**Figure 1:**
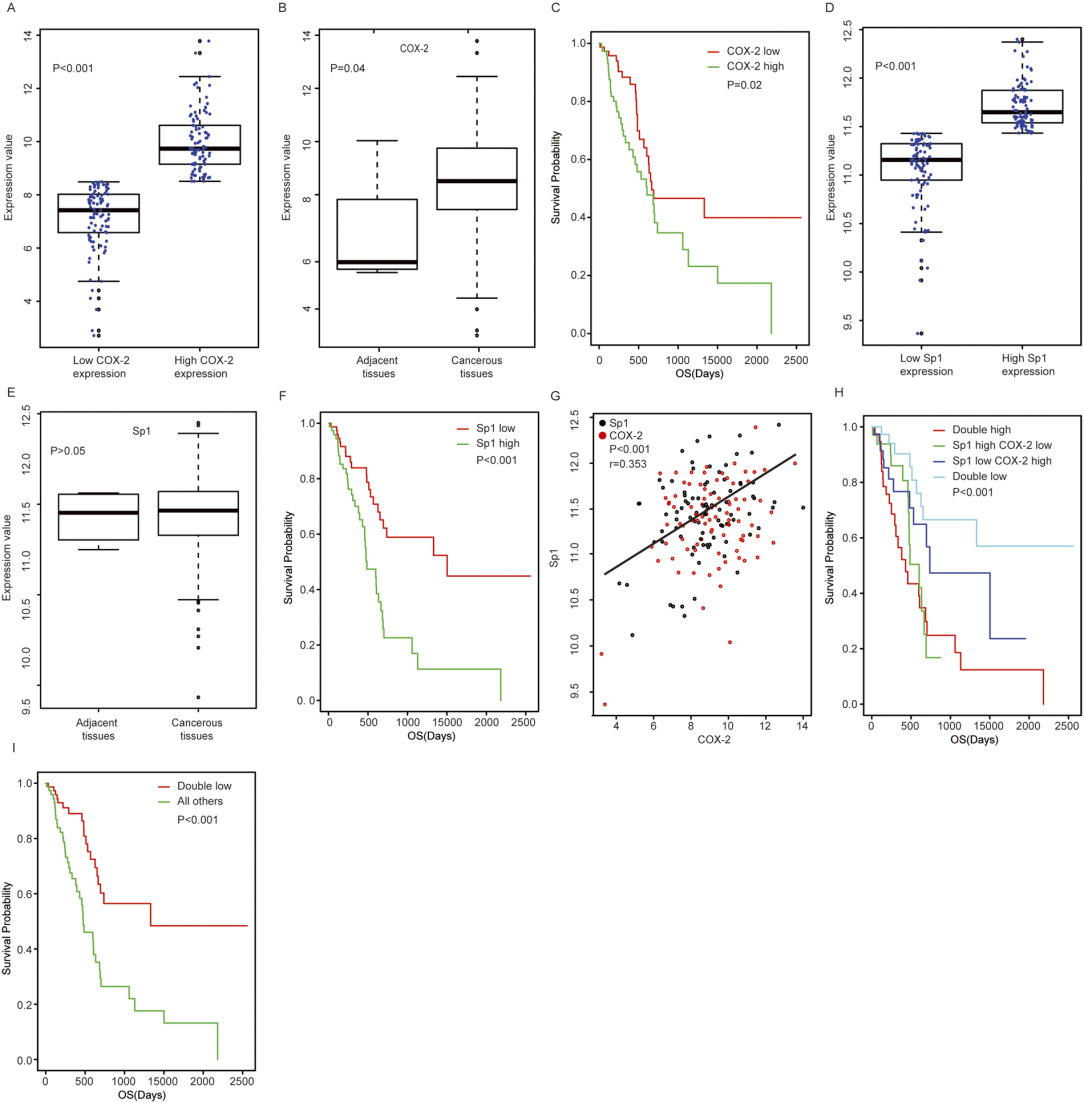
Correlated COX-2 and Sp1 expression in PDAC tissues. (**A**) The comparison of COX-2 expression between the high expression group and the low expression group. (**B**) The comparison of COX-2 expression between the cancerous tissues and the paired non-cancerous tissues. (**C**) Survival analysis based on COX-2 expression. (**D**) The comparison of Sp1 expression between the high expression group and the low expression group. (**E**) The comparison of Sp1 expression between the cancerous tissues and the paired non-cancerous tissues. (**F**) Survival analysis based on Sp1 expression. (**G**) Correlation analysis between COX-2 and Sp1. (**H,I**) Survival analysis based on Sp1 and COX-2.

To determine whether COX-2 was involved in the development of PDAC, we downloaded the PDAC RNA-seq data of from TCGA (http://cancergenome.nih.gov/) and analysed its expression. We found that COX-2 was expressed ubiquitously at different levels in all samples (Fig. 1A). In addition, a significant difference in COX-2 expression between the high expression group and the low expression group could also be observed (p < 0.001, Fig. 1A). Consistently, we also observed a significant difference in COX-2 expression between cancerous tissues and the paired non-cancerous tissues (p = 0.04, Fig. 1B). Moreover, a Kaplan-Meier assay showed that patients with high COX-2 expression tended to have a poor prognosis (p = 0.02, Fig. 1C). Collectively, these data suggested that COX-2 plays a critical role in PDAC development.

Meanwhile, the biological significance of Sp1 PDAC was also investigated. As shown in Fig. 1D, Sp1 is also expressed ubiquitously at different levels, with a significant difference between the high expression group and the low expression group (p < 0.001). However, no significant difference in Sp1 expression could be observed between the cancerous tissues and the paired non-cancerous tissues (p > 0.005, Fig. 1E). As with COX-2, a Kaplan-Meier assay showed that high Sp1 expression confers a poor survival for the patients (p < 0.001, Fig. 1F).

Sp1 is a ubiquitously expressed transcription factors that function extensively in cancer initiation and progression by binding to GC/GT boxes of target gene promoters to enhance transcription. Moreover, COX-2 is an inducible isoform of COX and its promoter is rich with GC/GT sequences. Thus, we postulated that Sp1 and COX-2 were correlated in PDAC patients. To verify this hypothesis, a correlation analysis was conducted and the data showed that Sp1 expression was positively correlated with COX-2 expression in PDAC patients (r = 0.353, p < 0.001, Fig. 1G and Table 1), with their simultaneous high expression conferring the worst prognosis among all patients (p < 0.001, Fig. 1H,I). Interestingly, we also observed that various patients showed high/low Sp1 expression but low/high COX-2 expression, which reflected the heterogeneity of the lethal disease. Of note, despite these patients accounted 44.8% of all the patients in total, they showed no significant influence on our conclusion that Sp1 was positively correlated with COX-2 in PDAC (r = 0.353, p < 0.001, Fig. 1G and Table 1). Altogether, these data showed that both COX-2 and Sp1 were upregulated, and they could synergistically promote PDAC progression.

**Table 1:** Sp1 was positively correlated with COX-2 in PDAC patients.

		Sp1 expression		Coefficient	P
		High	Low		
COX-2 expression	High	49 (27.5%)	40 (22.4%)	**0.353**	**<0.001**
	Low	40 (22.4%)	49 (27.5%)		

### Correlated expression of Sp1 and COX-2 in PDAC cell lines

**Figure 2:**
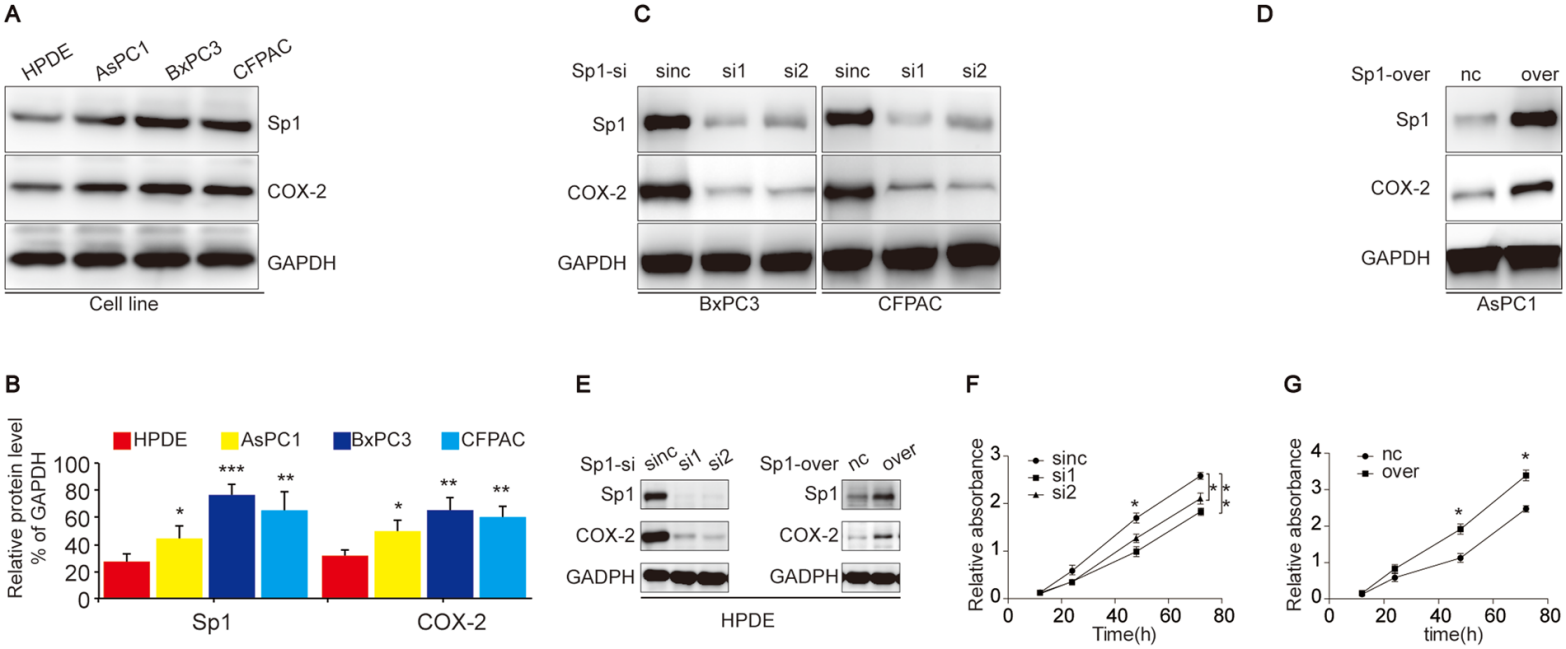
Correlated COX-2 and Sp1 expression in PDAC cell lines. (**A**) Detection of Sp1 and COX-2 expression in PDAC cell lines (BxPC3, CFPAC and AsPC1) and human pancreatic duct epithelial (HPDE) cells. (**B**) Relative densities of Sp1 and COX-2 expression are presented as Mean ± S.E (n = 3) of the fold change relative to GAPDH. *P < 0.05 and **P < 0.01. (**C**) Western blot showing COX-2 expression in BxPC3 and CFPAC cells upon Sp1 knockdown. (**D**) Western blot showing COX-2 expression in AsPC1 cells upon Sp1 overexpression. (**E**) Western blot showing Sp1 and COX-2 expression in HPDE cells upon Sp1 knockdown and overexpression. (**F,G**) CCK8 assay toward HPDE cells upon Sp1 knockout and overexpression.

We then investigated whether the positive correlation between Sp1 and COX-2 also existed in PDAC cells. First, we examined the expression of Sp1 and COX-2 in PDAC cell lines and human pancreatic duct epithelial (HPDE) cells. The data showed that PDAC cells have higher COX-2 and Sp1 expression than the HPDE cells (Fig. 2A,B). Then, Sp1 knockout or overexpressing cells were constructed accordingly. We found that upon Sp1 knockout or overexpression, the expression of COX-2 decreased or increased corresponding (Fig. 2C,D). Meanwhile, additional efforts indicated that Sp1 was also positively correlated with COX-2 in HPDE associating with enhanced proliferation capacity (Fig. 2E–G). These results showed that Sp1 was positively correlated with COX-2 in PDAC cells.

### Sp1 transcriptionally activates COX-2 expression in PDAC cells

**Figure 3:**
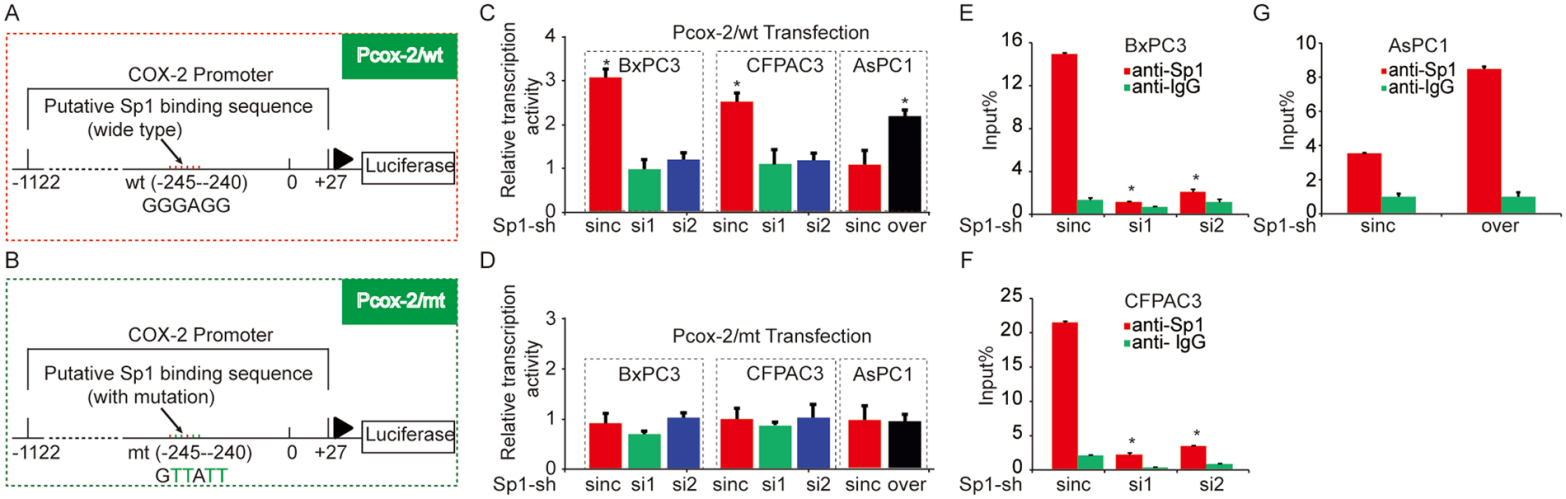
Sp1 transcriptionally activates COX-2 expression in PDAC cells. (**A**) Schematic diagram of luciferase reporter constructs with the wild-type COX-2 promoter sequence (Pcox-2/wt). (**B**) Schematic diagram of luciferase reporter constructs with the mutant COX-2 promoter sequence (Pcox-2/wt). The COX-2 promoter sequence was between positions −1122 and +27 from the transcriptional start site. (**C**) Luciferase activity of pCOX2/wt in PDAC cells. (**D**) Luciferase activity of Pcox-2/mt in PDAC cells. (**E,F**) ChIP assay conducted on Sp1 knockout and/or overexpression cells that also transfected with Pcox-2/wt using a specific anti-Sp1 antibody.

The data above prompted us to investigate the mechanism by which Sp1 interacts with COX-2 in PDAC cells. Since transcriptional activation was the major mechanism that links Sp1 to other factors, we hypothesized that this also applies to the correlation between Sp1 and COX-2 in PDAC. To test this, luciferase reporter constructs containing the wild-type human COX-2 promoter sequences (Pcox-2/wt) (Fig. 3A) and the same promoter with point mutations in putative Sp1 binding sequences at positions −240–245 (Pcox-2/mt) (Fig. 3B) were generated. The constructs were transfected into Sp1 knockdown and/or overexpression PDAC cells. Then the transcriptional activities of COX-2, indicated by luciferase activity, were measured. As shown in Fig. 3C, Sp1-knockout cells exhibited decreased transcription activities than the controls when they were transfected with Pcox-2/wt constructs. In contrast, Sp1-overexpressing cells exhibited higher transcription activity compared to the controls. Cells transfected with Pcox-2/mt constructs showed unchanged transcription activities compared to the control (Fig. 3D). These data indicated that the −240–245 sequences of the COX-2 promoter are essential for Sp1-driven transcription of COX-2 in PDAC cells.

To further confirm that Sp1 binds to the COX-2 promoter and activates its expression in PDAC cells, chromatin immunoprecipitation (ChIP) assays were conducted on Sp1 knockout and/or overexpression cells that also transfected with Pcox-2/wt using an anti-Sp1 antibody. qPCR, which aims to examine the endogenous COX-2 promoter sequences, on the fixed ChIP DNA products using specific COX-2 promoter primer sets showed a significant reduction of Sp1 binding to the COX-2 promoter in Sp1-knockdown cells compared to the control (Fig. 3E,F). In contrast, Sp1-overexpressing cells exhibited increased Sp1 binding to COX-2 promoter (Fig. 3G). Clearly, these data suggested that Sp1 could bind directly to COX-2 promoter to activate COX-2 expression in PDAC cells.

### Elevated COX-2 induces angiogenesis *in vitro*

**Figure 4:**
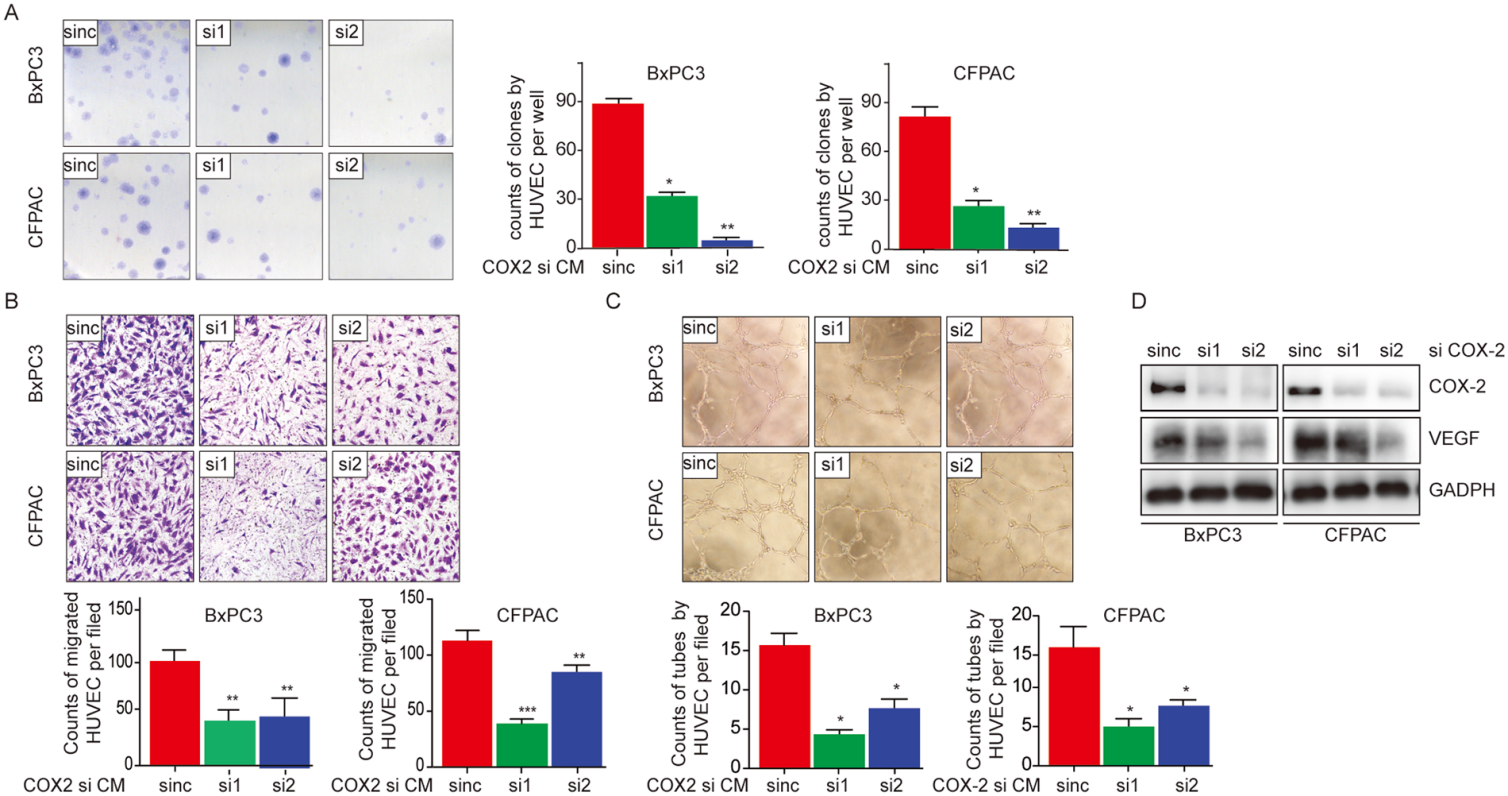
Elevated COX-2 contributes to angiogenesis in a process dependent on VEGF. (**A**) The clones formed by HUVECs when cultured in the conditioned media from COX-2 knockout cells. (**B**) The counts of migrated HUVECs when cultured in the conditioned media of COX-2 knockout cells. (**C**) The tubes formed by HUVECs when cultured in the conditioned media from COX-2 knockout cells. (**D**) VEGF expression in COX-2 knockout PDAC cells.

Considering that COX-2 is upregulated in cases of PDAC, we then investigated whether it could contribute to angiogenesis in this lethal disease. To this end, the conditioned media (CM) of COX-2 knockout cells were collected and used to culture HUVEC, and the migration, proliferation and tube formation of these cells were evaluated. We found that HUVEC cultured in the CM showed decreased proliferation (Fig. 4A), migration (Fig. 4B) and tube formation (Fig. 4C), suggesting that the angiogenic ability of HUVECs was decreased. Since VEGF is the most commonly studied pro-angiogenic factor, we then investigated its expression upon COX-2 deletion. As expected, VEGF expression decreased concomitantly (Fig. 4D). Altogether, these data showed that elevated COX-2 could promote angiogenesis in a manner at least partially dependent on VEGF in cases of PDAC.

### Aberrantly activated EGFR/p38-MAPK signalling functions as a major regulator of Sp1-induced COX-2 expression

**Figure 5:**
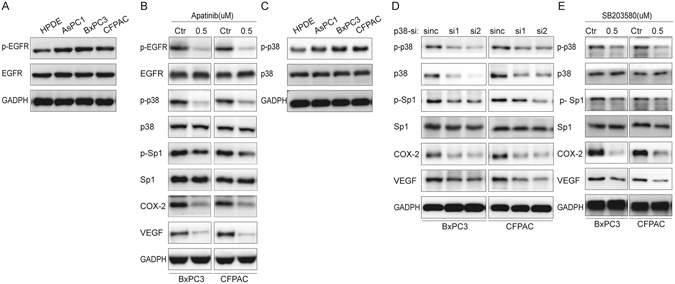
EGFR/p38-MAPK signalling functions upstream of Sp1-driven COX-2 induction. (**A**) The genetic profile of EGFR in PDAC cells as detected by Western blot. (**B**) p38, Sp1, COX-2 and VEGF expression upon pharmacological inhibition of EGFR with afatinib. (**C**) The genetic profile of p38 in PDAC cells as detected by Western blot. (**D,E**) The expression of Sp1, COX-2 and VEGF when p38 was inhibited using pharmacological (SB203580) or siRNA strategies.

The data presented above highlighted the biological significance of the Sp1/COX-2/VEGF axis in PDAC pathology, which prompted us to investigate the regulation of this pathway so as to reveal new therapeutic targets. Since phosphorylated Sp1 (p-Sp1) is the active form of Sp1 and aberrant functions of EGFR include hyperphosphorylation of downstream molecules^12, 13^, we hypothesized that over-activated EGFR functions upstream of the Sp1/COX-2/VEGF axis. To test this hypothesis, we examined the genetic profile of EGFR in PDAC cells. As shown in Fig. 5A, the activation of EGFR in PDAC cells was increased compared to that of HPDE cells. To further clarify the role of EGFR in this signalling pathway, EGFR activation was pharmacologically inhibited in BxPC3 and CFPAC using Afatinib. We found that the levels of p-Sp1, COX-2 and VEGF (Fig. 5B) decreased, while the levels of total Sp1 remained unchanged. These data suggested that EGFR functions upstream of Sp1/COX-2/VEGF signalling.

Considering that p38-MAPK was also activated in PDAC cells (Fig. 5C), and that the p38-MAPK signalling cascade was a major downstream EGFR signaling, we then assessed whether it is also involved in EGFR-triggered Sp1/COX-2/VEGF signalling using pharmacological inhibition and siRNA knockdown strategies. Our data showed that EGFR inhibition decreases the activation of p38-MAPK, accompanied by decreased Sp1 activation, COX-2 expression and VEGF expression (Fig. 5B). Consistently, we also observed that p38-MAPK inhibition leads to decreased Sp1 activation, COX-2 and VEGF expression (Fig. 5D,E). Altogether, these data showed that Sp1-induced COX-2 and VEGF expression was regulated by EGFR/p38-MAPK signalling.

## Discussion

**Figure 6:**
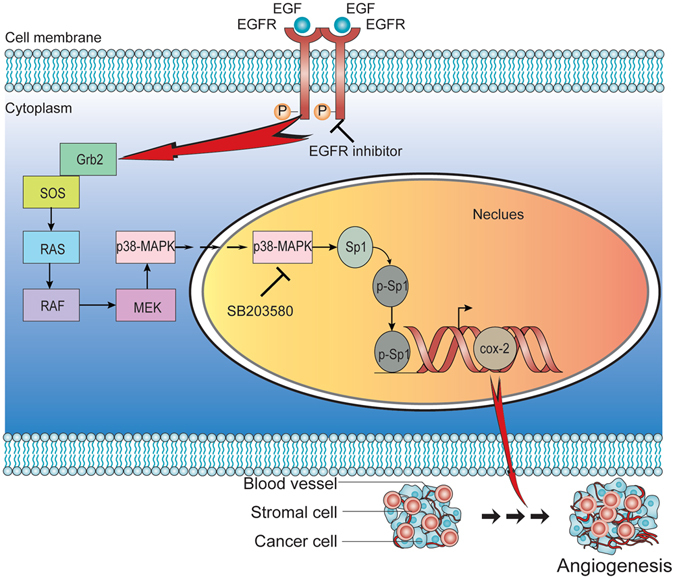
Schematic overview summarizing the functional significance and regulation of COX-2 in PDAC. Overactive EGFR phosphorylates Sp1 via a p38-MAPK signalling pathway. The highly activated Sp1 subsequently activates COX-2 transcription, leading to elevated COX-2 expression, which finally promotes the secretion of VEGF.

Our study clarified the functional significance and the regulation of COX-2 in PDAC. We showed that upregulated Sp1 in PDAC cells could transcriptionally activate COX-2, and that EGFR, together with its major downstream target p38-MAPK, is essential for the process. In addition, our data also revealed the pro-tumoural aspect of COX-2 in PDAC pathology relies on angiogenesis (Fig. 6).

It has been well established that COX-2 is elevated in most human malignancies and that its expression levels correlate with a poor prognosis^14–16^. In the present study, we identify elevated COX-2 in both clinical samples and PDAC cell lines. Consistently, the survival analysis suggested that a higher COX-2 expression confers a poorer prognosis than lower COX-2 expression. Considering the biological significance of COX-2 in malignancy, previous researches had evaluated the clinical usage of COX-2 inhibitors as an auxiliary approach to cancer prevention and management. For instance, celecoxib, a non-steroidal anti-inflammatory drug [NSAID] that selectively inhibits COX-2, was shown to reduce the extent of familial adenomatous polyposis^17, 18^, a condition resulting in colorectal cancers in 100% of cases^19^. In addition, Massimo and colleagues^20^ found that the combination of celecoxib and weekly paclitaxel is a safe and active regimen for pre-treated non-small cell lung cancer. With respect to PDAC, Suhail MD^21^ reported that the addition of celecoxib to gemcitabine and irinotecan substantially improves the response rate to PDAC treatment and life quality of the patients. Taken together, these data reveal that targeting COX-2 might also serve as an auxiliary approach to PDAC prevention and management. Based on this possibility, clarification of the regulation of COX-2 in cases of PDAC is of great clinical significance. As an inducible isoform of COX, COX-2 could respond to various cytokines and growth factors^22, 23^. Previously, multiple binding elements had been identified within the COX-2 promoter for TP53, NF-kb and other transcription factors^24, 25^. Structural analysis of this promoter suggested a high affinity for Sp1, as multiple GC sequences were identified within the promoter. Using luciferase reporter gene and ChIP assays, we provide the first evidence that Sp1 transcriptionally activates COX-2 expression, forming an Sp1/COX-2 signalling axis that has significance to PDAC.

**Figure 7:**
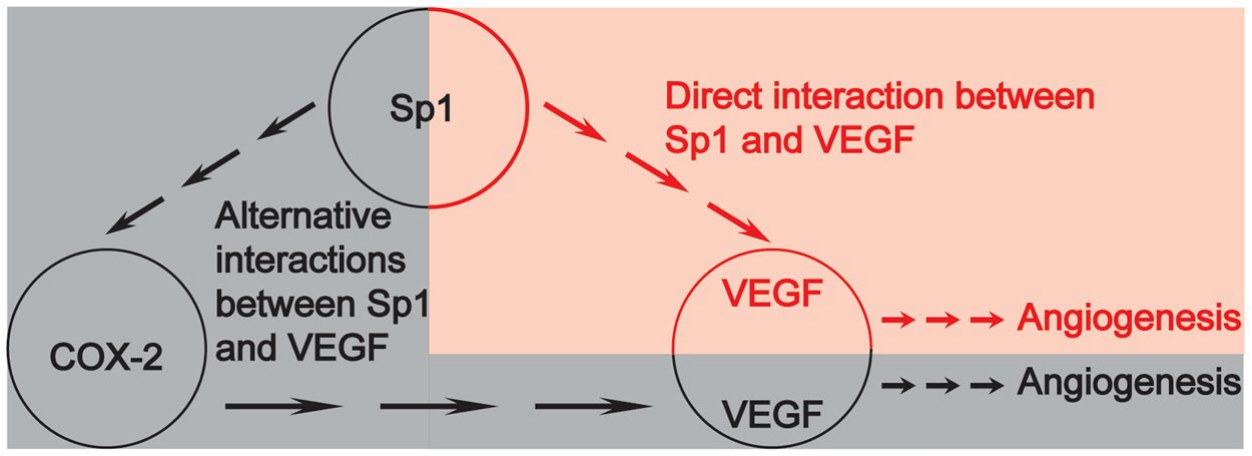
The complex signalling cascades between Sp1 and VEGF in PDAC. It has been reported previously that Sp1 can transcriptionally activate VEGF expression (shown in red). Our study shows that Sp1 can also promote VEGF expression via the upregulation of COX-2 expression (shown in black).

The pro-tumoural effects of COX-2 can be summarized as the promotion of neoplastic transformation and angiogenesis and protection against chemotherapy-induced apoptosis^26^. Using gene deletion strategies, we find that the pro-tumoural properties of COX-2 in PDAC cells rely on angiogenesis in a process dependent on VEGF. As we had previously shown that Sp1 could directly bind to the VEGF promoter and activate its expression^27^, our results add an alternative signalling pathway between Sp1 and VEGF in PDAC cells (Fig. 7), reflecting the complexity of this lethal disease.

Our data show that the pro-tumoural effects of COX-2 in PDAC rely on angiogenesis, suggesting that targeting angiogenesis might be an effective approach to counteracting the pro-tumoural effects of COX-2. Indeed, previous studies have addressed the functional significance of angiogenesis in PDAC. However, the fact that gemcitabine combined with bevacizumab, an antibody against VEGF, had no benefit in metastatic patients^28^ indicated that the role of angiogenesis in PDAC might not be as important as expected. Correspondingly, a recent study by Olive and colleagues^29^ proposed the concept of ‘vascular promotion’ for PDAC management. They showed in a mouse model that inhibition of the stroma sonic hedgehog (SHH) pathway could increase vascularization, leading to increased delivery of chemotherapeutic agents to tumours and greater anti-cancer efficacy. This concept is based on the theory that both human and genetically engineered models exhibit decreased vasculature due to accumulated desmoplasia. These studies were not contradictory but rather emphasized the complex and fast-changing pancreatic microenvironment.

EGFR is a tyrosine kinase receptor that plays a critical role in both physiological and pathological conditions. Upon activation, the receptors form dimers^12^ and undergo auto-phosphorylation, which subsequently triggers multiple downstream intracellular pathways leading to excess proliferation, invasion and metastasis, evasion of apoptosis and enhanced angiogenesis^12, 13^. Recent evidence has shown aberrant expression of EGFR in most human malignancies, such as lung cancer and breast cancer^30^. In clinical practice, EGFR inhibitors had improved the prognosis of several cancers. For example, the use of such drugs alone or in combination with other chemotherapeutic agents was shown to be efficient in prolonging the survival of patients diagnosed with lung, colorectal, head and neck cancers^31, 32^. In the current study, we show that EGFR signalling is sufficient to increase COX-2 expression via p38-MAPK-dependent Sp1 phosphorylation in PDAC cells. These findings support a recent randomized phase III trial that compared the combination of erlotinib and gemcitabine with gemcitabine alone in the treatment of PDAC, and showed that erlotinib significantly improved the response and survival rates of the patients^33^. Another irreversible EGFR inhibitor that was used in our study, afatinib, has also been reported to limit the growth of PDAC both *in vivo* ^34^ and *in vitro* ^35^, demonstrating that afatinib is also effective in inhibiting PDAC cells with aberrant EGFR activation.

In summary, we have showed the detailed function and regulation of COX-2 in PDAC. The results highlight the biological significance of EGFR signalling in PDAC, and support further investigation into whether the inhibition of Sp1 and COX-2 either alone or in combination with other well established targets could improve the prognosis of patients.

## Materials and Methods

### Bioinformatic Analysis

The mRNA expression levels of the patients were downloaded from The Cancer Genome Atlas (TCGA, https://cancergenome.nih.gov/). The website stored the R-Seq data of 183 pancreatic cancer patients, we excluded 5 samples due to the incomplete data. These data were public and could be freely downloaded. The expression value was used to represent the expression level of each gene. In the statistical analysis, the patients were divided into two groups, namely the high expression group and low expression group based on expression values. Specifically, their expression levels were arranged in a descending order, patients with the expression values above the median expression value were classified into the high expression group, while those with the expression values below the median expression value were classified into the low expression group.

### Cell Lines and Cell Culture

Human pancreatic duct epithelial (HPDE) cells and pancreatic cancer cell lines were purchased from the Shanghai Institute for Life Science, Chinese Academy of Sciences. All cells were cultured in RPMI 1640 supplemented with 10% foetal bovine serum (FBS, Gibco, Carlsbad, CA, USA) at 37 °C in a humidified atmosphere of 95% air and 5% CO_2_, and grown in a humidified atmosphere of air/CO_2_ (95%:5%). Cells with gene deletion or overexpression were cultured under the same conditions with 1.5 μg/mL puromycin (Sigma-Aldrich, St. Louis, MO, USA). Human umbilical vein endothelial cells (HUVEC) were purchased from PromoCell (PromoCell, Heidelberg, Germany) and cultured under the same conditions. For passage and experimental purposes, the cells were detached using trypsin-EDTA and resuspended in complete medium.

### Establishment of PDAC Cell Lines with Sp1 Short Hairpin RNAs (shRNAs)

The GV-248 Lentiviral RNAi Expression System (Genechem, Shanghai, China) was used to prepare lentivirus expressing shRNAs against human Sp1. The Sp1 knockout and overexpression plasmid was constructed by cloning the Sp1 cDNA into the lentiviral system. The targeting sequences were as follows: Sp1-Si1: 5′-GCAGTACCAATGGCAGCAATG-3′; Sp1-Si2: 5′-GCAGACCTTTACAACTCAA-3′. The scrambled sequence was 5′-TTCTCCGAACGTGTCACGT-3′. The sequences used to generate Sp1 overexpressing cells were reported previously^36^. Polyclonal cells with puromycin resistance were selected for subsequent experiments. The protocol for transfection was described previously^37^.

### Transfection

Cells were plated in 6-well plates and transfected with 3 μL of siRNA in the presence of 4 μl RNAiMax (Invitrogen) according to the manufacturer’s instructions. Two different siRNAs and a control siRNA were purchased from GenePharma Technologies (Shanghai, China). Gene silencing and overexpression effects were confirmed by Western blot analysis at 48 hours post-transfection.

### Endothelial Migration Assay

For endothelial migration assays, conditioned media (CM) were harvested from PDAC cells cultured in 60-mm plates in RPMI-1640 without FBS for 24 hours. The media were added to the bottom chambers of 24-well tissue culture plates in triplicate. HUVEC (40,000 cells) were seeded into Boyden chambers (8 μm pore size with polycarbonate membrane). The chambers were then inserted into a transwell apparatus (Costar, Cambridge, MA, USA). Cells were allowed to migrate for 12 h, and then fixed and stained in 0.3% crystal violet for 30 mins. After rinsing with PBS, the migrated cells were subjected to microscopic inspection.

### Tube Formation Assay

For endothelial tube formation assays, the CM was added to 2000 HUVEC in sextuplicate wells of Matrigel-coated 96-well plates (BD Biosciences, Franklin Lakes, NJ, USA). Cells were grown in a humidified atmosphere of air/CO_2_ (95%:5%) at 37 °C overnight. The degree of tube formation was evaluated using an inverted microscope.

### Sphere Formation Assay

For sphere formation assays, single cell suspensions were washed twice using serum-free phosphate buffer solution (PBS) and plated in 6-well ultralow attachment plates (Corning, Steuben County, New York, USA) at a density of 200 cells in the conditional media at 37 °C in a humidified atmosphere of 95% air and 5% CO_2_ for 21 days. Spheres were fixed with 4% buffered formalin for 15 minutes and stained with 1% crystal violet for 30 minutes. The plates were gently washed with PBS and dried for microscopic sphere evaluation under a phase contrast fluorescence microscope (Nikon, ECLIPSE 80).

### Western Blot Analysis

The procedures for Western Blot Analysis had been stated previously^38^. In Brief, cells were washed three times with cold PBS and lysed on ice in RIPA buffer with the protease inhibitor PMSF (Beyotime Biotechnology, China). Protein concentrations were determined by BCA assay (Beyotime Biotechnology, China). A total of 20 μg protein was separated by 10% SDS-PAGE and electro-blotted onto NC membranes using a semi-dry blotting apparatus. After blocking in 3% bovine serum albumin (BSA), the membranes were incubated with primary antibodies overnight at 4 °C. The membranes were washed and incubated with secondary antibodies for 1 h at room temperature on a shaker. The protein bands were visualized using a commercially available enhanced chemiluminescence kit (Thermo Scientific, Hudson, NH, USA). GAPDH and β-Actin were used as controls. The primary antibodies used in this study include Sp1, p-Sp1, COX-2, p38, p-p38, EFFR, p-EGFR (CST, Beverly, MA, USA); VEGF (Abnova, Taipei, Taiwan); β-Actin and GAPDH (Santa Cruz Biotechnology, CA, USA). The quantitation of the blots using image J as described previously^39^.

### COX-2 Promoter/Luciferase Constructs

The wild-type COX-2 promoter sequence from positions −1,122 to +27 relative to the transcriptional start site was generated using a site-directed mutagenesis kit (Agilent, Roseville City, CA), and labelled as Pcox-2/wt. The mutant construct of the COX-2 promoter (labelled as Pcox-2/mt) was created similarly with putative Sp1 binding sequences changed from GGGAGG to GTTAAC. These sequences were inserted upstream of the luciferase open reading frame in a GV238 vector containing the neomycin resistance gene.

### Luciferase Reporter Gene Assay

Luciferase reporter assays were performed using pooled cells as described previously^40^. Briefly, the constructs were introduced into PDAC cells as described above. Luciferase activity was quantified using a dual luciferase assay system (Promega, Madison, WI) as described previously^41^.

### Chromatin Immunoprecipitation (ChIP) Assay

ChIP assay was performed using a ChIP Assay Kit (Merck Millipore, Germany) according to the manufacturer’s instructions. In brief, Sp1-overexpression and/or knockdown cells that also transfected with Pcox-2/wt were cross-linked by adding formaldehyde to a final concentration of 1% when they grown at 90% confluency in a 150-mm dish, and rocked for 10 minutes at room temperature. Immunoprecipitation was performed using antibodies against Sp1 to pull down Sp1-associated DNA. The DNA, which was mainly endogenous COX-2 promoter sequences, was purified using a QIAquick spin column and eluted in 50 μl/column of 10 mm Tris, pH 8.0. After that, the COX-2 promoter DNA associated with Sp1 was determined by hot semi quantitative PCR using the following primer pair: (forward) 5′-cgggcaaagactgcgaagaagaaa-3′ and (reverse) 5′-aaccaagcccatgtgacgaaatgac-3′. This generated a 280 base pair product that encompasses the Sp1 putative binding site at positions −245 to −240. The forward primer was labelled using T4 polynucleotide kinase and [γ-32P] ATP, and approximately 0.5 pmol of hot primer were added to a PCR reaction containing 10 pmol of each cold primer. PCR conditions were as follows: 94 °C × 30 seconds, 58 °C × 30 seconds, and 72 °C × 45 seconds for 20 cycles. A portion of DNA recovered from an aliquot of sheared chromatin was used as the “input”. Normal rabbit serum was used as a negative control.

### Statistical Analysis

All statistical analysis was performed with SPSS statistical software (version 21.0, SPSS Inc., Chicago, IL, USA). Associations between different categorical data were analysed using χ2 statistics. The probability of survival with different groups was calculated using the Kaplan-Meier method, and statistical significance was analysed using the log-rank test. All statistics were two-sided, and a p value of less than 0.05 was considered statistically significant.
